# Relationship between pan-immune- inflammation value and in major cardiovascular and cerebrovascular events in stable coronary artery disease patients undergoing on-pump coronary artery bypass graft surgery

**DOI:** 10.1186/s13019-024-02691-1

**Published:** 2024-04-17

**Authors:** Ahmet Dolapoglu, Eyup Avci

**Affiliations:** 1 Department of Cardiovascular Surgery, Balikesir University Faculty of Medicine, Balikesir, Turkey; 2 Department of Cardiology, Balikesir University Faculty of Medicine, Balikesir, Turkey

**Keywords:** Pan-immune-inflammation value, Major cardiovascular and cerebrovascular events, Stable coronary artery disease, Coronary artery bypass graft

## Abstract

**Background:**

In this study, we aimed to evaluate the association of pan-immune-inflammation value (PIV) with major cardiovascular and cerebrovascular events (MACCE) in stable coronary artery disease patients undergoing on-pump coronary artery bypass graft (CABG) surgery.

**Methods:**

We retrospectively analyzed data from 527 patients who underwent on-pump CABG surgery for stable coronary artery disease between June 2015 and December 2020. Patients were categorized into two groups based on MACCE development. PIV levels were calculated from blood samples taken on admission. PIV was calculated as [neutrophil count (×10^3^/µL)×platelet count (×10^3^/µL))×monocyte count (×10^3^/µL)]/lymphocyte count (×10^3^/µL). The primary endpoint was long-term major cardiovascular and cerebrovascular events (MACCE) at a median follow-up of 4.6 years.

**Results:**

Of the included patients, 103 (19.5%) developed MACCE. PIV was higher in patients with MACCE compared to those without (470.8 [295.3-606.8] vs. 269.8 [184.3-386.4], *p* < 0.001). Multivariate analysis showed a significant positive association between PIV and MACCE (HR: 1.326, 95%CI:1.212–1452, *p* < 0.001). The cut-off value for the PIV in the estimation of MACCE was 368.28 ( AUC: 0.726 with 69% sensitivity, 71% specificity, *p* < 0.001).

**Conclusion:**

This study shows a significant link between high PIV levels and MACCE in stable coronary artery disease patients undergoing on-pump CABG surgery. Our findings suggest that PIV may be a valuable, routinely available, and inexpensive marker for identifying patients at increased risk of MACCE.

## Introduction

Cardiovascular disease has become one of the leading causes of morbidity and mortality worldwide [1]. Coronary artery bypass graft (CABG) surgery is a highly effective intervention for improving cardiac blood flow and reducing the risk of myocardial infarction [2]. The rate of major cardiovascular and cerebrovascular events (MACCE) was found as 19.7 at 5 years after surgery in a previous study by Johannesdottir et al. [3]. SYNTAX trial showed that 5-year MACCE was %26.9 [4]. The freedom-from-MACCE survival for CABG patients at 5 years in the Arterial Revascularization Therapies Study (ARTS) was 78% [5].

It is the role of inflammatory markers in surgical interventions, suggesting a potential link between inflammation and perioperative complications [6, 7]. The markers including neutrophil-lymphocyte ratio (NLR), monocyte-lymphocyte ratio (MLR), platelet-lymphocyte ratio (PLR), and systemic immune-inflammation index (SII) are well-established indicators of inflammation and have been studied in various cardiovascular conditions, including acute coronary syndrome and acute myocardial infarction [8, 9]. Moreover, it has been shown that the association of preoperative different inflammatory markers, including NLR, MLR, PLR, and SII with poor outcomes in patients who underwent CABG surgery [10–12].

The pan-immune-inflammation value (PIV) introduction is intriguing, as it encompasses a broader range of blood cell populations, providing a more comprehensive reflection of systemic inflammation and immunity. PIV has recently been proposed as a robust predictor of clinical outcomes in advanced colorectal cancer and acute coronary syndrome patients [13, 14].

In this study, we aimed to show the association of PIV with MACCE in patients who underwent CABG.

## Methods

We retrospectively enrolled 527 consecutive patients with stable coronary artery disease, who electively underwent an on-pump CABG surgery from June 2015 to December 2020. The patients were followed for 12 months after their operations. Clinical characteristics, laboratory findings, surgical details, and postoperative outcomes were obtained from the patient’s chart. The patients in critical preoperative state, patients with chronic kidney disease on dialysis, patients who had active endocarditis, patients with concomitant valve disease requiring concomitant surgical intervention, patients with a previous history of cardiac surgery, and patients who underwent urgent CABG were excluded from the study. According to their MACCE at follow-up, the patients were divided into two groups: MACCE (+) and MACCE (-). The researchers abided by the principles of good clinical practice and the Declaration of Helsinki, and The local ethics committee approved the study (number of ethics committee: 2022-E.190,976). The requirement for informed consent was waived as part of the study design. All of the subjects’ details had been de-identified.

All CABG procedures were carried out on-pump, with a cross-clamped aorta. After general anaesthesia, a median sternotomy was made. Cardiopulmonary bypass was initiated after heparin administration at the proper level of activated clotting time. To protect the heart muscle during the procedure, cold blood cardioplegia was used and conducted with multiple doses through antegrade and retrograde ways of administration. While the left internal mammary artery was utilized to graft the left anterior descending artery (LAD) as much as possible, the great saphenous vein was used as a graft for all other coronary vessels. Complete revascularization was achieved in all patients. Information regarding the MACCE was obtained from a national electronic database, an institutional electronic database, and phone calls with patients/relatives.

All patients were examined using echocardiography before their surgical procedures. A modified Simpson`s method was used for the assessment of left ventricular end-diastolic/systolic volumes and ejection fractions. The biochemical panel, complete blood count, and coagulation panel of all patients were analyzed at their admission. PIV was calculated as [neutrophil count (×10^3^/µL)×platelet count (×10^3^/µL))×monocyte count (×10^3^/µL)]/lymphocyte count (×10^3^/µL) [11]. Systemic-immunoinflammatory index (SII) was calculated as neutrophil count to lymphocyte count ratio x platelet count.

### Endpoints

The primary endpoint was long-term major cardiovascular and cerebrovascular events (MACCE) at a median follow-up of 4.6 years. MACCE was a composite of all-cause mortality defined as any cause of death, myocardial reinfarction defined as STEMI or non-ST-segment elevation myocardial infarction, target vessel revascularization defined as any repeat revascularization in the epicardial vessel (main branch or side branches), and cerebrovascular events which included the occurrence of new neurological deficits (such as stroke or transient ischemic attack) confirmed through radiological imaging. The composite endpoint was assessed by time to the first event. Clinical follow-up information of the patients was obtained by review of clinic visits or by telephone. Clinical visits of the patients were made face to face or by telephone at 3-month intervals in the first year after surgery, and at 6-month intervals after the first year. The mortality or MACCE data for patients who were lost to follow-up were confirmed using the National Death Records, and National Social Security Institution.

### Statistical analysis

Continuous variables were provided as mean ± standard deviation and categorical variables were shown as the number of patients, with the percentage of the total number. Either the Student’s t-test or the Mann-Whitney U test was used to compare values between the two groups, as appropriate. The Chi-squared test was used to compare categorical variables. Univariate and multivariate logistic regression analyses were performed to search for predictors of long-term MACCE. The predictive values of NLR, monocyte, platelet, SII, PIV, multivariable model, and multivariable model plus PIV were estimated by the areas under the receiver operating characteristic curve (ROC). We determined the cut point value for PIV as 368.28 according to the ROC curve to show its predictiveness of PVI for MACCE risk. Then we divided the patients into 2 groups as low (< 368.28, *n* = 332) and high risk (≥ 368.28, *n* = 195) according to this value. We performed propensity score matching to create a matched dataset (low-risk vs. high-risk) and the covariates including age, chronic obstructive pulmonary disease, hypertension, smoking, peripheral artery disease. and vasopressor usage were considered for balance between low-risk and high-risk groups. Matching based on propensity scores produced 194 patients in low-risk group, and 195 patients in high-risk group. The cumulative incidence of the primary and secondary end-points was estimated by the Kaplan–Meier. Two-sided *p*-values < 0.05 were considered statistically significant. All statistical analysis was carried out with SPSS version 26 (SPSS Inc., Chicago, IL, USA) and R software.

## Results

The median follow-up time was 4.6 (3.7–5.7) years. The mean age of the patients included in the study was 65 ± 7.9 years and 25% of the patients were female. Demographic, clinical, and angiographic characteristics of all the patients of the groups were presented in Table 1. Patients in the MACCE group were older. There were no differences between the groups in terms of the history of hypertension. However, diabetes mellitus, chronic obstructive pulmonary disease, and peripheral artery disease were more common in patients with MACCE compared with those without MACCE (Table 1). IABP use was higher in the MACCE group than in the non-MACCE group (6% vs. 2%, *p* = 0.043).

**Table 1:** Demographic, clinical, and angiographic characteristics of patients

Variable	MACCE (-) (*n* = 424)	MACCE (+) (*n* = 103)	*p*-value
Age (years)	63.4 ± 8.8	67.3 ± 7.5	< 0.001
Female gender n (%)	105 (25)	26 (25)	0.920
Diabetes mellitus n (%)	129 (30)	48 (47)	0.002
Hypertension n (%)	91 (22)	30 (29)	0.097
COPD n (%)	38 (9)	24 (24)	< 0.001
PAD n (%)	20 (5)	17 (17)	< 0.001
Dyslipidemia n (%)	91 (22)	16 (16)	0.180
Smoking n (%)	65 (15)	20 (19)	0.312
IABP usage n (%)	9 (2)	6 (6)	0.043
Vasopressor usage n (%)	81 (19)	22 (21)	0.605
LVEF (%)	49.0 ± 7.3	46.0 ± 8.1	< 0.001
CPB time (min)	56.6 ± 13.3	57.2 ± 12.9	0.700
Cross-clamp time (min)	36.5 ± 10.4	37.8 ± 10	0.234
Outcomes			
Mortality n (%)	0 (0)	49 (49)	< 0.001
Repeated revascularization n (%)	0 (0)	53 (53)	< 0.001
Myocardial reinfarction n n (%)	0 (0)	35 (35)	< 0.001
Cerebrovascular events n (%)	0 (0)	14 (14)	< 0.001

**Abbreviations:** MACCE; major cardiovascular and cerebrovascular events, LVEF; left ventricular ejection fraction, CPB – cardiopulmonary bypass, IABP: intra-aortic balloon pump, COPD; chronic obstructive pulmonary disease, PAD; peripheral artery disease

Laboratory findings are presented in Table 2. Haemoglobin level was significantly lower in patients with MACCE than those without MACCE (12.3 ± 1.6 vs. 12.9 ± 1.4, *p* < 0.001). The patients in the MACCE group had higher levels of SII [859 (585–1173) vs. 627 (489–830), *p* < 0.001] and PVI [470.8 (295.3-606.8) vs. 269.8 (184.3-386.4), *p* < 0.001]

**Table 2:** Laboratory findings of patients

Variable	MACCE (-) (*n* = 424)	MACCE (+) (*n* = 103)	*p*-value
BMI	22.5 ± 2.0	22.2 ± 2.4	0.117
Hemoglobin (mg/dl)	12.9 ± 1.4	12.3 ± 1.6	< 0.001
Creatinine (mg/dl)	0.94 ± 0.29	0.92 ± 1.9	< 0.001
SII* (x10^9^/L)	627 (489–830)	859 (585–1173)	< 0.001
WBC (×10^3^/µL)	8.1 ± 1.7	8.7 ± 2.0	0.001
Neutrophil (×10^3^/µL)	5.35 ± 1.4	5.95 ± 1.6	< 0.001
Lymphocyte(×10^3^/µL)	2.17 ± 0.6	2.11 ± 0.7	0.447
Monocyte (×10^3^/µL)	0.45 ± 0.16	0.52 ± 0.2	< 0.001
Platellet (×10^3^/µL)	266.35 ± 69.9	300.93 ± 82.6	< 0.001
NLR	2.6 ± 1.0	3.2 ± 1.6	< 0.001
PIV*	269.8 (184.3-386.4)	470.8 (295.3-606.8)	< 0.001

**Abbreviations**: MACCE; major cardiovascular and cerebrovascular events BMI; body mass index, WBC; white blood cell, SII;systemic-immunoinflammatory index, NLR; neutrophil to lymphocyte ratio, PIV; Pan-Immune-Inflammation Value

* Comparison was made using the Mann-Whitney U test at *p*<0.05, and these values were described by a median with an interquartile range (25th and 75th percentile)

**Fig. 1:**
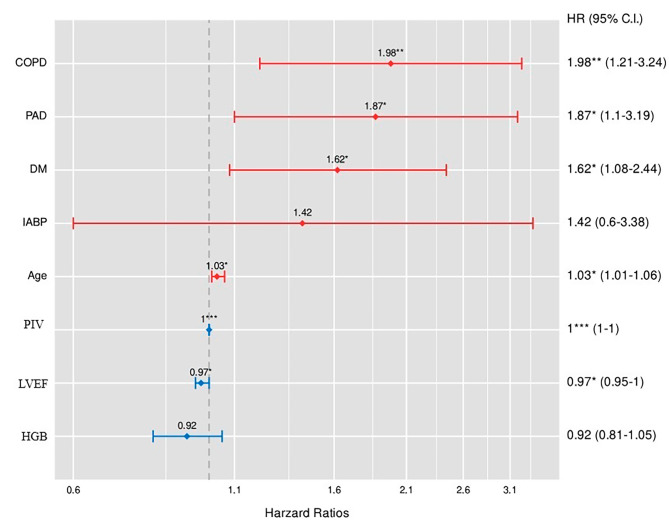
The long-term predictors of major cardiovascular and cerebrovascular events (MACCE). COPD; chronic obstructive pulmonary disease, PAD; peripheral artery disease, DM; diabetes mellitus, IABP; intra-aortic balloon pump, LVEF; left ventricular ejection fraction, HGB; haemoglobin, PIV; Pan-Immune-Inflammation Value

**Fig. 2:**
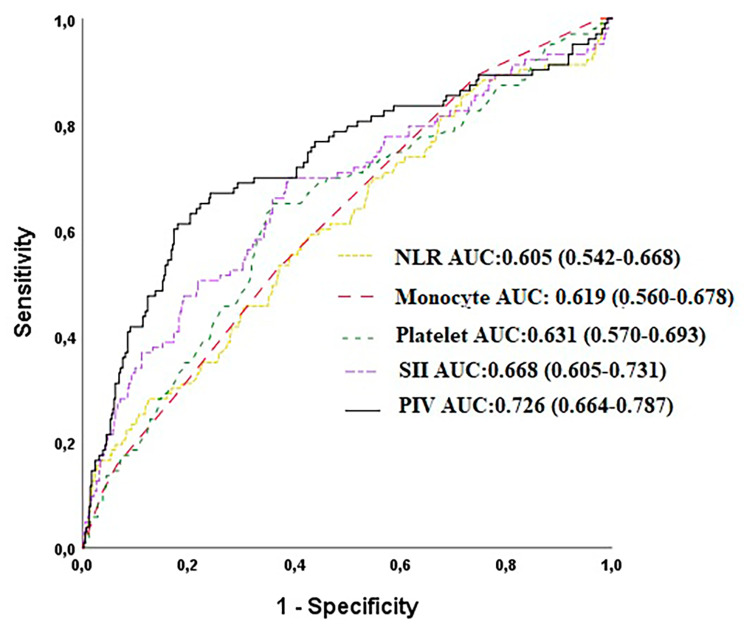
Receiver operating characteristic (ROC) curves for the neutrophil to lymphocyte ratio( NLR), monocyte, platelet, systemic-immunoinflammatory index (SII ), and Pan-Immune-Inflammation Value (PIV) for predicting major cardiovascular and cerebrovascular events (MACCE).

**Fig. 3:**
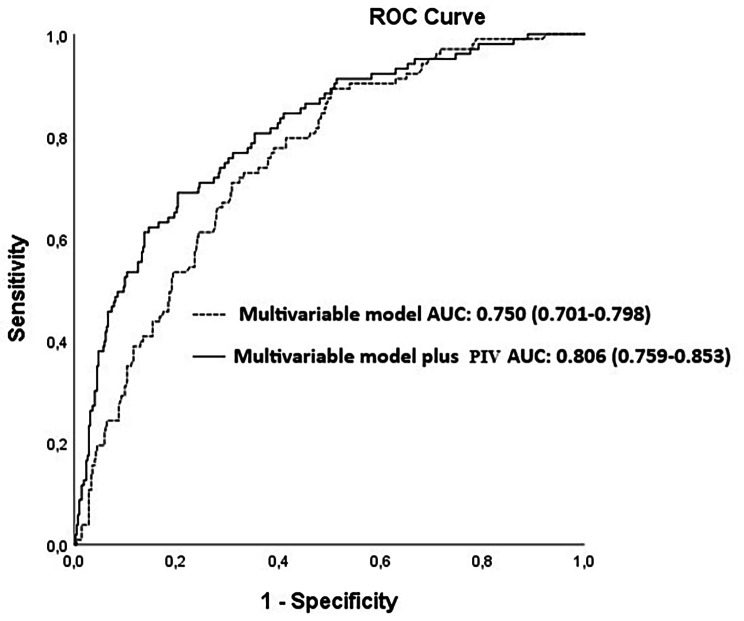
Receiver operating characteristic (ROC) curves for the multivariable model, multivariable model plus Pan-Immune-Inflammation Value (PIV) for predicting major cardiovascular and cerebrovascular events (MACCE).

In multivariate analysis, PIV was an independent predictor of MACCE (HR: 1.326, 95%CI:1.212–1452, *p* < 0.001, Fig. 1). The cut-off value for the PIV in the estimation of MACCE was 368.28 ( AUC: 0.726 with 69% sensitivity, 71% specificity, Fig. 2). The value of PIV in predicting MACCE was better than NLR alone ( AUC:0.726 vs. 0.605, z = 4.740, difference *p* < 0.001) and PLT alone (AUC:0.726 vs. 0.631, z = 3.106, difference *p* = 0.002). Moreover, we found that PIV had higher accuracy in predicting MACCE compared with SII alone (AUC: 0.726 vs. 0.668, z = 3.053, *p* = 0.002, Fig. 2). Also, the combination of PIV with the multivariable model including age, LVEF, peripheral artery disease, chronic obstructive pulmonary disease, and diabetes mellitus significantly improved the prognostic performance of the multivariable model (multivariable model vs. multivariable plus PIV; AUCs: 0.806 vs. 0.705, z = 2.874, *p* = 0.004, Fig. 3).

**Fig. 4:**
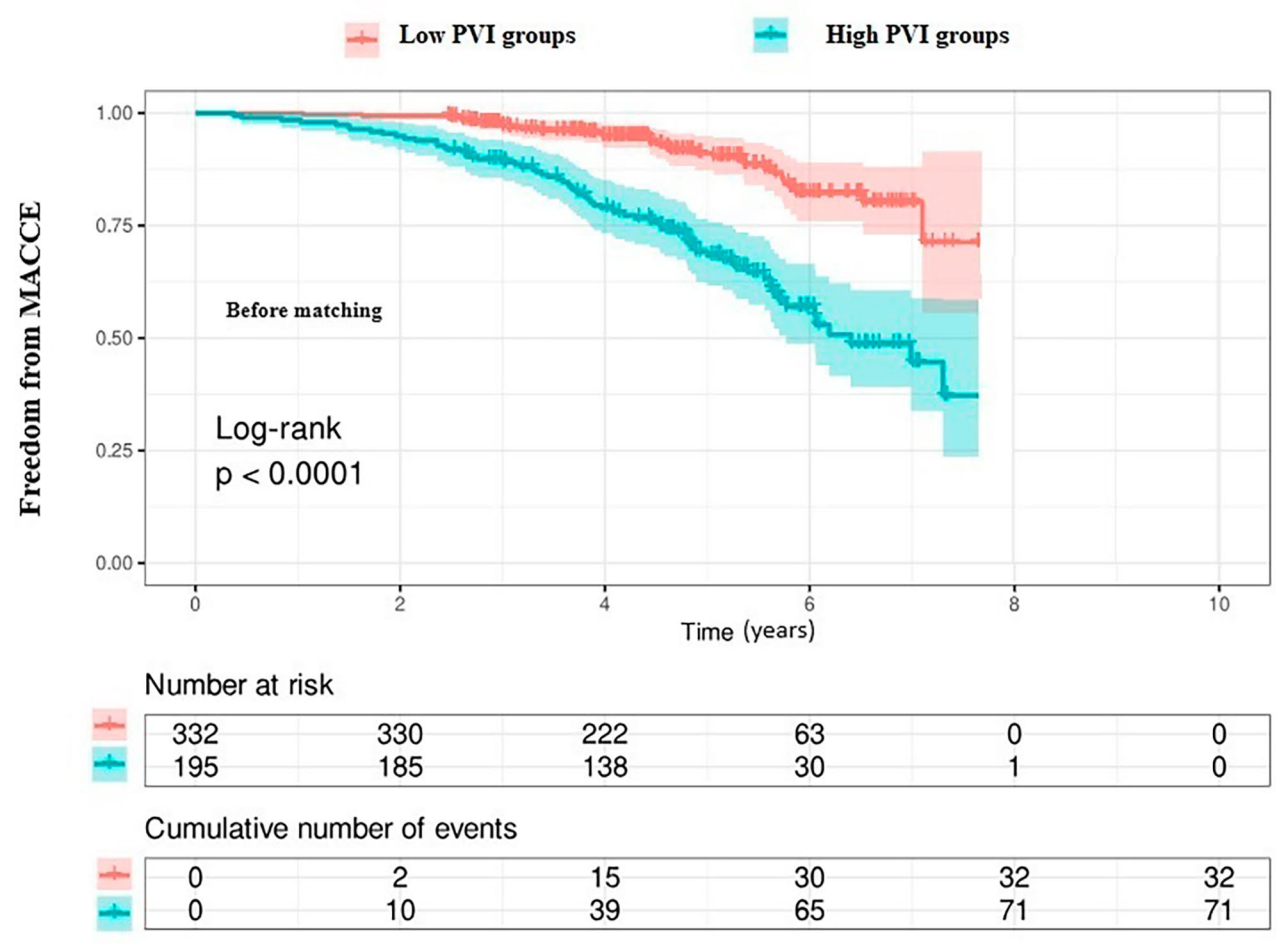
Kaplan–Meier estimate of the cumulative incidence of the major cardiovascular and cerebrovascular events (MACCE ) according to Pan-Immune-Inflammation Value (PIV) before matching

**Fig. 5:**
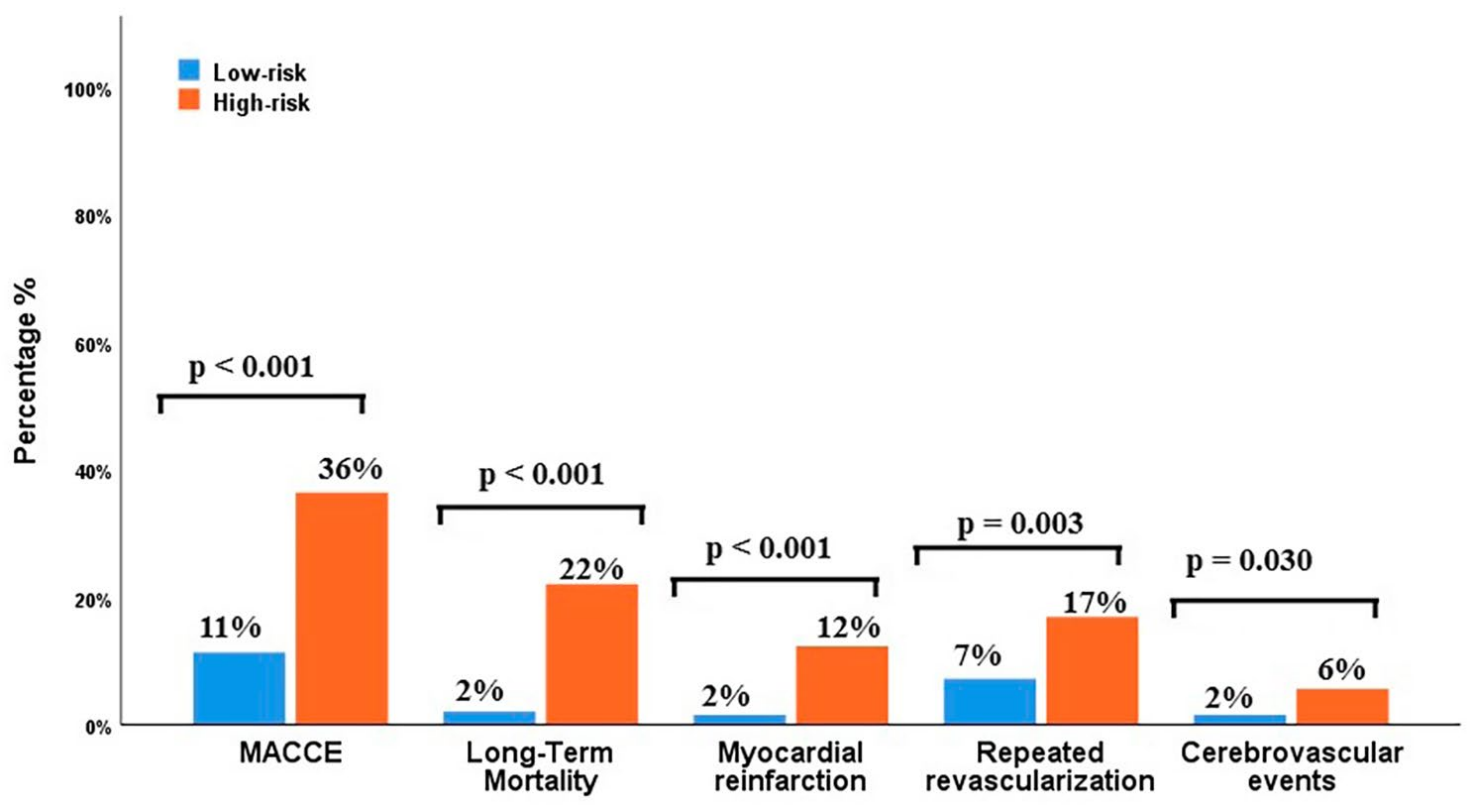
Major cardiovascular and cerebrovascular events (MACCE ) in matching group

**Fig. 6:**
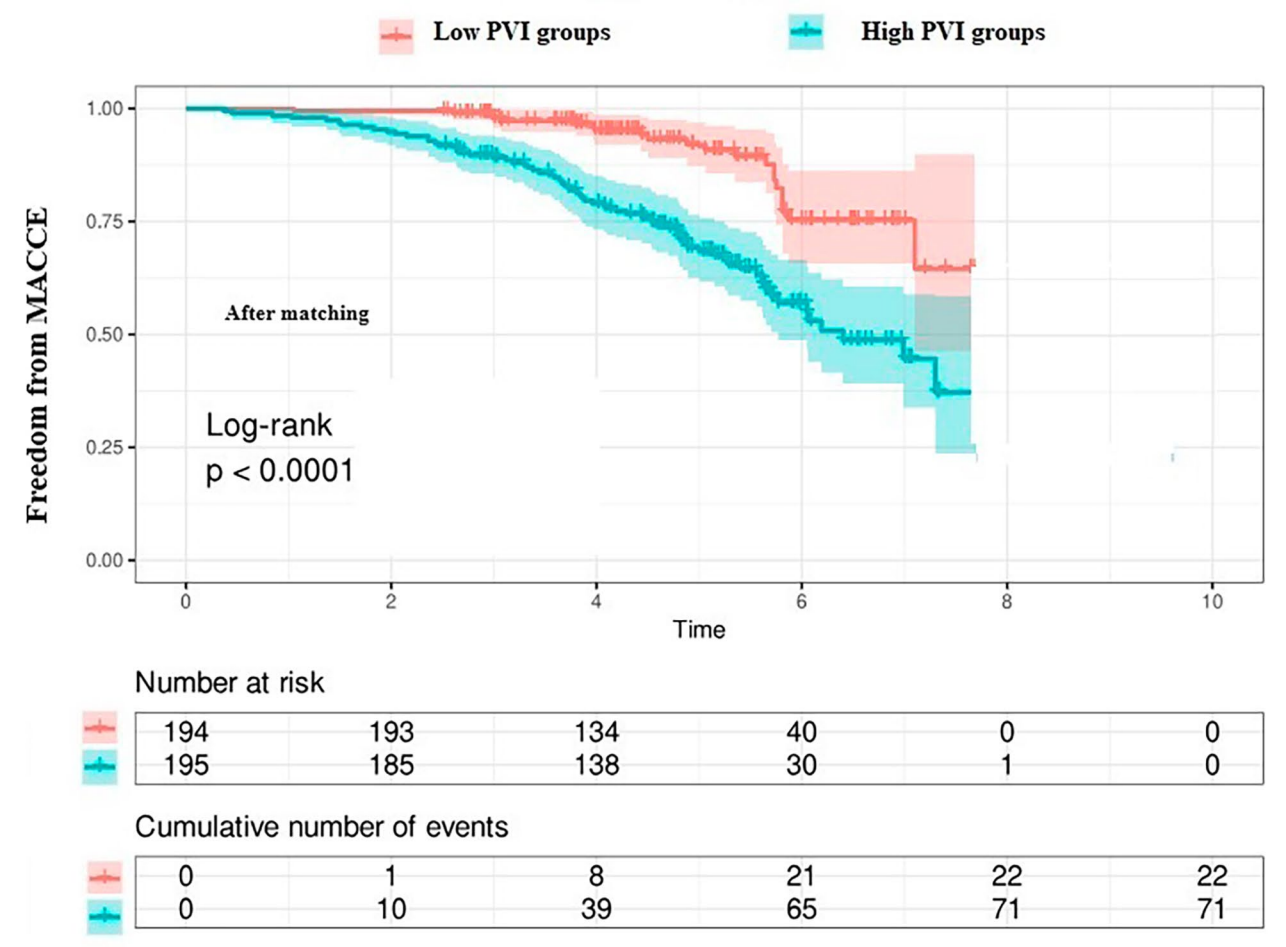
Kaplan–Meier estimate of the cumulative incidence of the major cardiovascular and cerebrovascular events (MACCE ) according to Pan-Immune-Inflammation Value (PIV) after matching

The patients were divided into 2 subgroups based on the cut-off value of PIV: low (< 368.28) and high subgroups (≥ 368.28). In subgroup analysis, the MACCE rate was higher in patients with high than low group (38% vs. 9%, *p* < 0.001). Kaplan-Meier event-free survival curves according to a cut-off value of PIV before matching were shown in Fig. 4. Matching based on propensity scores produced 194 patients in the low-risk group, and 195 patients in the high-risk group (Tables 3 and 4) and showed that the MACCE rate was high in the high-risk groups *(*36% vs. 11%, *p* < 0.001, Table 3; Fig. 5). The rate of MACCE after matching is provided in Fig. 6.

**Table 3:** Demographic, clinical, and angiographic characteristics of patients

Variable	Before matching			After matching		
	Low-group (*n* = 332)	High-group (*n* = 195)	*p*-value	Low-group (*n* = 194)	High-group (*n* = 195)	*p*-value
Age (years)	63.7 ± 8.5	64.9 ± 9.1	< 0.001	64.0 ± 8.1	64.9 ± 9.2	0.313
Female gender n (%)	81 (24)	50 (26)	0.750	58 (30)	50 (26)	0.349
Diabetes mellitus n (%)	109 (33)	68 (35)	0.632	81 (42)	68 (35)	0.163
Hypertension n (%)	65 (20)	56 (29)	0.016	32 (17)	56 (29)	0.004
COPD n (%)	31 (9)	31 (16)	0.024	20 (10)	31 (16)	0.103
PAD n (%)	18 (5)	19 (10)	0.061	12 (6)	19 (10)	0.195
Dysplidemia n (%)	69 (21)	38 (20)	0.721	48 (25)	38 (20)	0.212
Smoking n (%)	45 (14)	40 (21)	0.036	34 (18)	40 (21)	0.453
IABP usage n (%)	8 (2)	7 (4)	0.432	2 (1)	7 (4)	0.093
Vasopressor usage n (%)	55 (17)	48 (25)	0.024	31 (16)	48 (25)	0.034
LVEF (%)	48.7 ± 7.6	47.9 ± 7.5	0.204	48.2 ± 6.7	47.9 ± 7.5	0.638
CPB time (min)	56.8 ± 13.9	56.6 ± 12.0	0.869	55.9 ± 14.4	56.6 ± 12.0	0.627
Cross-clamp time (min)	36.6 ± 10.8	37.0 ± 9.5	0.656	35.6 ± 10.3	37.0 ± 9.5	0.177
Outcomes						
Mortality n (%)	6 (2)	43 (22)	< 0.001	4 (2)	43 (22)	< 0.001
Repeated revascularization n (%)	20 (6)	33 (17)	< 0.001	14 (7)	33 (17)	0.003
Myocardial reinfarction n n (%)	11 (3)	24 (12)	< 0.001	3 (2)	24 (12)	< 0.001
Cerebrovascular events n (%)	3 (1)	11 (6)	0.001	3 (2)	11 (6)	0.030
One-year MACCE n (%)	0 (0)	1 (1)	0.192	0 (0)	1 (1)	0.318
3- year MACCE n (%)	9 (3)	19 (10)	0.001	4 (2)	19 (10)	0.001
5-year MACCE n (%)	12 (4)	35 (18)	< 0.001	7 (4)	35 (18)	< 0.001

**Abbreviations**: LVEF; left ventricular ejection fraction, CPB – cardiopulmonary bypass, IABP: intra-aortic balloon pump, COPD; chronic obstructive pulmonary disease, PAD; peripheral artery disease, MACCE; major cardiovascular and cerebrovascular events

**Table 4:** Laboratory findings of patients

Variable	Before matching			After matching		
	Low-group (*n* = 332)	High-group (*n* = 195)	*p*-value	Low-group (*n* = 332)	High-group (*n* = 195)	*p*-value
BMI	22.5 ± 2.1	22.5 ± 2.1	0.968	22.6 ± 2.2	22.5 ± 2.1	0.602
Hemoglobin (mg/dl)	12.8 ± 1.4	12.7 ± 1.6	0.518	12.7 ± 1.4	12.7 ± 1.6	0.830
Creatinine (mg/dl)	0.90 ± 0.26	0.90 ± 0.30	0.771	0.90 ± 0.27	0.90 ± 0.3	0.919
SII* (x10^9^/L)	550 (434–665)	944 (752–1177)	< 0.001	566 (438–686)	944 (752–1177)	< 0.001
WBC (×10^3^/µL)	7.6 ± 1.6	9.2 ± 1.8	0.001	7.6 ± 1.3	9.2 ± 1.8	< 0.001
Neutrophil (×10^3^/µL)	4.91 ± 1.3	6.42 ± 1.3	< 0.001	4.92 ± 1.0	6.42 ± 1.3	< 0.001
Lymphocyte(×10^3^/µL)	2.23 ± 0.6	2.10 ± 0.7	0.002	2.20 ± 0.6	2.10 ± 0.7	0.011
Monocyte (×10^3^/µL)	0.40 ± 0.1	0.58 ± 0.2	< 0.001	0.40 ± 0.1	0.58 ± 0.2	< 0.001
Platellet (×10^3^/µL)	251.42 ± 56.3	310.04 ± 84.7	< 0.001	253.64 ± 62.1	310.04 ± 84.7	< 0.001
NLR	2.3 ± 0.7	3.5 ± 1.5	< 0.001	2.3 ± 0.7	3.5 ± 1.5	< 0.001
PIV*	221.6 (167.0-282.3)	510.8 (417.2-606.8)	< 0.001	221.8 (169.5-297.4)	510.8 (417.2-606.8)	< 0.001

**Abbreviations**: BMI; body mass index, WBC; white blood cell, SII; systemic-immuno inflammatory index, NLR; neutrophil to lymphocyte ratio, PIV; Pan-Immune-Inflammation Value

## Discussion

This study showed that PIV was an independent predictor of long-term MACCE in these patients. Patients with high PIV were at high risk of developing MACCE.

Atherosclerosis is known to involve inflammatory processes, and several studies have shown a connection between inflammation, activation of inflammatory cells, and the occurrence of coronary artery disease (CAD), which is a result of atherosclerosis affecting the coronary arteries [15–18]. Certain parameters derived from routine blood tests, specifically the complete blood count, can provide insights into the inflammatory status. Neutrophils, a type of immune cell associated with innate immunity, play a significant role in inflammation-induced vascular damage [19]. When activated during inflammation, neutrophils produce reactive oxygen species that can damage the vascular wall [20]. Neutrophils also form extracellular traps (NETs), which can contribute to thrombosis and coagulation [21]. Activated neutrophils promote platelet activation and deposition [22]. Platelets, in turn, aid in recruiting inflammatory cells to atherosclerotic lesions and release various mediators that contribute to the inflammatory environment within the arteries [23]. Monocytes, another type of immune cell, are pro-inflammatory and contribute to atherosclerosis development [24]. They take up oxidized lipids and form foam cells, which are a core component of atherosclerotic plaques. The secretion of proinflammatory cytokines, enzymes, and growth factors by monocytes further exacerbates plaque formation and can lead to plaque destabilization [25]. The role of lymphocytes, specifically T-cells, B-cells, and natural killer T (NK T) cells, is complex and can be either protective or pro-atherogenic [26]. T-cells, especially Th1 cells, can promote endothelial dysfunction and lipid accumulation in macrophages, contributing to atherosclerosis. Regulatory T cells, on the other hand, have anti-inflammatory effects that can limit atherogenic processes. B-cells produce antibodies that may have both protective and detrimental effects depending on the type of antibody [26]. It has been shown that a low lymphocyte count has been linked to worse cardiovascular outcomes in individuals with coronary artery disease [27].

By considering multiple biomarkers, we may be able to build more accurate prediction models for outcomes like disease progression, response to treatment, or overall survival compared with a single biomarker. Researchers have shown that higher NLR value was associated with an increase in both short and long-term mortality in patients who underwent CABG [10, 26–28]. Another study found there was a relationship between MLR and worse outcomes in on-pump CABG surgery patients [11]. Similar to the results of the above studies, patients with MACCE in our study had high NLR and monocyte values. The Systemic Inflammatory Index (SII) is a novel inflammatory index that takes into account the levels of three different types of blood cells: neutrophils, lymphocytes, and platelets [29]. These cells are important components of the immune response and are often used as indicators of inflammation and immune system activity in the body [29]. The use of the SII provides a more comprehensive view of the immune and inflammatory status compared to analyzing each cell type separately. An elevated SII value was associated with a higher level of systemic inflammation and has been linked to mortality in patients treated with cardiac surgery [12] as found in the presented study.

PIV integrates various components of the immune system, specifically neutrophil, platelet, monocyte, and lymphocyte counts, derived from a complete blood cell count (CBC) [30]. The PIV appears to be a composite value that takes into account the relative proportions or counts of these immune cell types in the bloodstream. Each of these cell types may have different roles in the immune response and could be indicative of different aspects of inflammation. Combining information from all four cells can provide a more accurate and nuanced representation of the inflammatory state in a given individual. A recent study has shown that PIV was associated with both short and long-term mortality in acute myocardial infarction patients [14]. The presented study showed that PVI was an independent predictor of long-term mortality in patients treated with on-pump CABG surgery.

The patients with higher levels of inflammatory markers before undergoing cardiac surgery might have a more extensive atherosclerotic burden or more active coronary artery disease. In other words, the elevated inflammatory markers may be a reflection of the chronic risk factors these patients have, potentially making their cardiovascular conditions more severe.

The present study has several limitations. It is a single-centre, retrospective study, in which controls are often recruited by convenience sampling, and are thus not representative of the general population and prone to selection bias. Also, it is prone to recall bias or misclassification bias. It is subject to confounding (other risk factors may be present that were not measured). In the presented study we cannot determine causation, only association because of the retrospective nature of the study. Moreover, we only analyzed patients with stable complex coronary disease. We aimed to form the most homogenous study group, therefore, the acute coronary syndrome was an exclusion criterion. Patients with concomitant diseases requiring surgical intervention were not included due to already proven worse results of combined procedures. Furthermore, we did not measure the dynamic change of PIV in these patients. Because of these limitations, prospectively designed, larger, multicentre studies are mandatory to verify the mechanism behind the association of PIV and increased MACCE rate in patients who underwent CABG surgery.

## Conclusions

This study showed that preoperative PIV levels were associated with the risk of recurrent MACCE in stable coronary artery disease patients who underwent CABG surgery. Further studies are needed to determine whether baseline PIV measurements can guide risk stratification, treatment decisions, and postoperative management strategies for individuals with coronary artery disease requiring CABG surgery.

## Data Availability

The data supporting this study’s findings are available from the corresponding author upon reasonable request.
